# Oxaliplatin-loaded nanoemulsion containing *Teucrium polium* L. essential oil induces apoptosis in Colon cancer cell lines through ROS-mediated pathway

**DOI:** 10.1080/10717544.2022.2096711

**Published:** 2022-07-10

**Authors:** Waad A. Al-Otaibi, Sahar M. AlMotwaa

**Affiliations:** Department of Chemistry, College of Science and Humanities, Shaqra University, Shaqra, Saudi Arabia

**Keywords:** Oxaliplatin, *Teucrium polium* L. essential oil, Combination therapy, nano-delivery system, Reactive oxygen species, flow cytometry, ROS-mediated mitochondrial apoptosis, nanoemulsion

## Abstract

Oxaliplatin (Oxa)-associated adverse side effects have considerably limited the clinical use of the drug in colon cancer therapy. Mutant p53 has diverse mutational profiles in colon cancer, and it influences the potencies of various chemotherapeutic drugs, including Oxa. Thus, it would be highly beneficial to identify an alternative therapeutic strategy that not only reduces the toxicity of Oxa, but also exerts a synergistic effect against colon cancers, regardless of their p53 profiles. The present study was aimed at preparing and optimizing *Teucrium polium* L. essential oil nanoemulsion (TPO-NANO) and investigating its effect on the sensitivity of colon cancer cells with differences in p53 status (HCT116 wild-type and HT-29 mutant-type) to Oxa. The viability of treated cells was determined and the combination index (CI) was calculated. Morphological changes were determined under inverted microscopy, while percentage apoptosis was assayed using flow cytometry. Intracellular ROS and the protein levels of p53 and Bax were measured. The colony-forming potential of treated cells was determined using colony assay. The size of TPO-NANO was markedly increased from 12.90 ± 0.04 nm to 14.47 ± 0.53 nm after loading Oxa (*p* ≤ 0.05). The combination (Oxa + TPO-NANO) produced a synergetic effect in HCT116 and HT-29, with CI of 0.94 and 0.88, respectively. Microscopic examination and flow cytometric analysis revealed that cells treated with Oxa + TPO-NANO had a higher percentage of apoptosis than cells exposed to monotherapy. Cumulatively, Oxa exerted an apoptotic effect on wild or mutant p53 colon cancer cells when combined with TPO-NANO, through a mechanism involving ROS-mediated mitochondrial apoptosis.

## 1. Introduction

Colon cancer has been increasing progressively in recent years, and it has been ranked third in cancer-associated death worldwide (Therachiyil et al., 2020). Although surgical resection is considered the main therapeutic option at the early stages of the disease, chemotherapy with Oxa remains the cornerstone in the treatment of patients with advanced colon cancer (Chen et al., 2018). Oxaliplatin (Oxa), which is considered the standard platinum anticancer agent, acts by inhibiting DNA synthesis via intra-strand cross-link between guanine base pairs in DNA, in addition to inducing apoptosis in cancer cells (Vivek et al., 2014). In mammalian cancer cells, apoptosis is stimulated through extrinsic or intrinsic pathways after exposure to Oxa. The extrinsic pathway is initiated by death receptor 5 (DR5) on the cell surface, resulting in the activation of caspase 8. In contrast, the intrinsic pathway is characterized by the central role of mitochondria in the initiation of the apoptosis-associated caspase cascade. Anti- and pro-apoptotic members of the Bcl-2 family play pivotal roles in the intrinsic apoptosis pathway (Bhardwaj et al., 2016). After exposure to apoptotic stimuli, the pro-apoptotic proteins (e.g., Bax) are transferred to the outer membrane of mitochondria, leading to the release of apoptotic factors from mitochondria, caspase activation and apoptotic cell death. In addition, p53, a tumor suppressor gene, directly regulates Bax, and both proteins have important roles in cellular response to chemotherapy. It is known that p53 plays are crucial in DNA repair, apoptosis, and cell cycle arrest in response to external cellular stress. However, 40-50% of colon cancers have a mutant-type p53 (Rattanawong et al., 2018). Mutant p53 protein is responsible for invasion, proliferation, migration, angiogenesis and drug resistance (Muller & Vousden, 2013). It has been demonstrated that resistance to anticancer agents (including Oxa) is linked to mutated p53 (Arango et al., 2004). However, the clinical applications of Oxa for colon cancer treatment are limited due to adverse side effects, an example of which is neurotoxicity which leads to peripheral neuropathy (Peng et al., 2022).

Cancer cells are more sensitive to additional oxidative stress than nonmalignant cells, due to a higher oxidative stress baseline. As consequence, cancer cells are very sensitive to agents that elevate ROS levels (Farah et al., 2016). In the last decade, research has focused on the use of natural products in combination with chemotherapeutics. It has been well documented that natural compounds are potent ROS generators which interfere with cellular signaling pathways (Rezacova, 2022; Wu et al., 2022). *Teucrium polium* L. belongs to the Lamiaceae family. It is a perennial shrub 20-50 cm high, with 3-cm-long linear-shaped leaves (Bahramikia & Yazdanparast, 2012). *Teucrium polium* L. is one of the most fragrant plants fairly distributed throughout the country of Saudi Arabia. The fragrance is related to aromatic compounds in the plant (Migahid, 1978). Essential oil of *Teucrium polium* L. plant (TPO) exerts anticancer activity which is related to the presence of different kinds of secondary metabolites such as τ-cadinol, β-eudesmol, myrcene, α-phellandrene and caryophyllene oxide. These bioactive compounds have been reported to exert antiproliferative effects through induction of oxidative stress by elevating ROS levels, and through stimulation of cell death via mitochondria-mediated apoptosis (Al-Otaibi & AlMotwaa, 2022; Su et al., 2013; Kotawong et al., 2018; Bomfim et al., 2013; Bai & Tang, 2020; Lin et al., 2013; Ding & Chen, 2018; Patel & Thakkar, 2014). Thus, combination therapy with Oxa and ROS-inducing agent could further enhance the sensitivity of colon cancer cells to Oxa. Moreover, there is need to evolve a novel, nontoxic and effective therapy free from p53 chemoresistance. However, the use of large-scale TPO is not feasible due to the hydrophobicity and volatility of the bioactive components of the oil (Esmaeili & Asgari, 2015). Thus, previous studies reported several attempts at incorporating the oil into different delivery systems (Mansouri et al., 2021; Almasi et al., 2021; Upadhyay et al., 2021; Hassan et al., 2021; Al-otaibi, 2021). A lot of attention has been focused on the use of nano-emulsions to deliver the oils by forming water-based products so as to enhance the solubility of the insoluble components (Donsì & Ferrari, 2016). These nano-delivery systems are composed of nanodroplets of oil dispersed in a water system stabilized with surfactant and co-surfactant, with size below 200 nm (McClements & Rao, 2011).

To the best of our knowledge, there are no extant studies on the anticancer activity of TPO when incorporated into a nano-system. The present study was aimed at formulating, optimizing, and characterizing a nano-delivery system based on TPO, using the Box-Behnken design method (BBD). Moreover, the anticancer activity of oxaliplatin incorporated into nano-emulsion containing TPO oil was evaluated *in vitro* in a p53 wild-type human colon cancer cell line HCT116 and a p53 mutant human colon cancer cell line, HT-29.

## 2. Materials and methods

### 2.1. *Teucrium polium* L. Oil

The fragrant oil of *Teucrium polium* L. (local name: *Jaad*) was extracted in our previous work (Al-Otaibi & AlMotwaa, 2022) from the aerial parts of the plant growing in Horimlaa (Northwest of Riyadh, KSA) at the flowering stage. The chemical constituents of the extracted oil (TPO) were determined with gas chromatography-mass spectroscopy (GC-MS) in our previous work. The major constituents obtained from TPO were τ-cadinol (25.58%), α-fenchene (20.09%), β-eudesmol (11.76%), β-myrcene (8.02%), γ-cadinene (5.22%), α-phellandrene (3.11%) and caryophyllene oxide (2.48%).

### 2.2. Chemicals and reagents

Phosphate buffered saline (PBS), RPMI-1640 medium, Dulbecco’s modified eagle medium (DMEM), trypsin-EDTA (0.25%), penicillin-streptomycin, and heat-inactivated fetal bovine serum (FBS) were obtained from Thermo Fisher scientific, Gibco (Waltham, MA, USA). Tween 80 (T80), propylene glycol (PG) and other chemicals were obtained from Al Shafei for Scientific and Medical Equipment, Est (Jeddah, KSA). Reactive oxygen species (ROS), Annexin V-FITC apoptosis detection kit, enzyme-linked immunosorbent assay (ELISA) and colorimetric kits for proteins determination were purchased from Abcam Inc., Cambridge, UK.

### 2.3. Cell culture

Two different human colon cancer cell lines: HT-29 and HCT116 were obtained from American Type Culture Collection (ATCC). The cells were cultured in their optimum media (DMEM or RPMI-1640) containing 10% FBS and 1% (v/v) penicillin-streptomycin in a humified 5-% CO_2_ incubator at 37 °C. The culture medium for each cell line was replaced with a fresh medium every 2 days until 90% confluence was attained. Thereafter, the confluent cells were detached using 2 ml trypsin, and incubated for 5 min.

### 2.4. Preparation of TPO-NANO formulations

Nano-emulsion of *Teucrium polium* L. essential oil (TPO-NANO) was produced with high-pressure homogenization method using Tween 80 (T80) and propylene glycol (PG) as surfactant and co-surfactant, respectively. Different proportions of distilled water, TPO and T80 were mixed in a 5-ml Pyrex screw-cap test tube and heated to 60 °C, with constant mixing using Vortex mixer F202A0173 (VELP SCIENTIFICA, Italy) at a speed of 3000 rpm for 10 min. Thereafter, PG was added to the final mixture, and the mixture was continuously vortexed at 3,000 rpm for 15 min. The resultant mono-phase emulsion was subjected to 5 rounds of homogenization at 1000 rpm in a high-pressure homogenizer (AVESTIN EMULSIFLEX C5; Avistin, Inc., Ottawa, ON, Canada). The resultant transparent/semi-transparent solution was subsequently used for further analysis.

### 2.5. Particle size measurements

The mean droplet diameter and polydispersity index (PDI) of the prepared formulation were measured at 21 ± 2.5 °C using a dynamic light scattering instrument (Zetasizer Nano series, Malvern Instruments, Ltd., Malvern Worcestershire, UK).

### 2.6. FTIR analysis

Fourier Transform Infrared (FTIR) spectroscopy was used to determine the interaction between Oxa and TPO-NANO. Measurement was carried out at 25^°^ C from 600 to 4000 cm^−1^ resolution using an FTIR spectrophotometer (Thermo, Nicolet iS 50).

### 2.7. Optimization of TPO-NANO with Box-Behnken statistical design

Box-Behnken design (BBD) method was used for optimization of TPO-NANO formulation. Using BBD, the impact of production factors such as T80 (%), TPO (%) and PG:T80 ratio on output variables (droplet diameter and PDI) were evaluated. Fifteen different experimental runs composed of three independent variables and their three coded levels are illustrated in Table S1. For each input variable, the experimental range was chosen based on preliminary experiments and screening studies. Data for output variables were collected in each run and analyzed using Minitab®^19^ statistical software. The quadratic model consisting of a three-factor, three-level design was generated for each response variable from the software, and depicted in the following equation:

$$\text{R}={\text{I}}_{0}+{\text{ I}}_{\text{1}}\text{A}+{\text{I}}_{\text{2}}\text{B}+{\text{I}}_{\text{3}}\text{C}+{\text{I}}_{\text{12}}\text{AB}+{\text{I}}_{\text{13}}\text{AC}+{\text{I}}_{\text{23}}\text{BC}+{\text{I}}_{\text{11}}{\text{A}}^{\text{2}}+{\text{I}}_{\text{22}}{\text{B}}^{\text{2}}+{\text{I}}_{\text{33}}{\text{C}}^{\text{2}}$$

where R is the output variable associated with each level of factor combination, I_0_ is an intercept, I_1_ to I_3_ are the regression coefficients, while A, B and C are coded levels for input variables. Accordingly, the corresponding output variables were predicted based on the model generated by the software. Then, the optimized formulation was chosen based on PDI and minimum values of the droplet diameter. Subsequently, the optimum formulation was selected, prepared, and the output variables of the optimized formula were experimentally evaluated. Using the following equation, the absolute error was assessed based on the variation between experimental and predicted values of the output variables:

$$\mathrm{Error}\;(\%)=\frac{\text{Experimental value}-\text{Predicted value}}{\text{Experimental value}}\times \;100$$

### 2.8. Preparation of Oxa + TPO-NANO and Oxa-NS

Oxaliplatin (Actavis, New Zealand) was incorporated into the optimized formulation of TPO-NANO to produce the loaded formulation (Oxa + TPO-NANO) at a concentration of 40 ug/ml (100 uM). Similarly, a stock solution of free Oxa (Oxa-NS) was prepared at a concentration of 40 ug/ml (100 uM) in physiological saline [0.9% (w/v) NaCl].

### 2.9. Determination of cytotoxicity

The cytotoxic effects of Oxa-NS, TPO-NANO, and their combination on HCT116 and HT-29 cell lines were evaluated in terms of mitochondria functionality using 3-(4,5-dimethyl thiazolyl)2,5-diphenyl-tetrazolium bromide (MTT) assay. In this assay, the cells were plated in 96-well plates, each at a density of 10^4^ cells/well, and incubated in a 5% CO_2_ atmosphere for 24 h at 37 °C, before treatment. Then, the cells were treated for 24 h with different concentrations of the formulations which were serially diluted 2-fold in the appropriate culture medium. The optical densities (OD_s_) of the dissolved formazan crystals were read spectrophotometrically at 570 nm. Wells containing only culture media were considered as blank, while wells containing cells in culture media were untreated control. Experiments for each sample were performed in triplicate. Values of percentage vitality for HCT116 and HT-29 cell lines treated with various concentrations of the formulations were calculated using the following equation:

$$\mathrm{Vitality}(\%)=\left(\frac{\;\left.\mathrm{Abs}\text{ of }\mathrm{treated}\;\mathrm{well}\;–\;\mathrm{Abs}\text{ of }\mathrm{blank}\right.}{\left.\mathrm{Abs}\text{ of }\mathrm{control}\;(\mathrm{untreated})–\;\mathrm{Abs}\text{ of }\mathrm{blank}\right.}\times \;100\right)$$

To determine if Oxa + TPO-NANO produced better therapeutic results on HCT116 and HT-29 cells than the individual components, the data were analyzed with Compusyn software (ComboSyn, Inc., Paramus, NJ. USA). Values of combination index (CI) were calculated for constant-ratio combination (1:1), thereby allowing for evaluation of synergistic (CI> 1), additive (CI = 1) and antagonistic (CI< 1) effects.

### 2.10. Determination of effects of treatments on cell morphology

The morphological alterations in HCT116 and HT-29 cancer cells were determined following treatment for 24 h with combined formulation and individual drugs at concentrations equivalent to IC_50_ values under the same incubation conditions indicated earlier. Thereafter, the cellular morphologies of treated and untreated cells were examined under an inverted microscope (Olympus Southall, UK) at x200 magnification.

### 2.11. Assay of intercellular ROS concentrations

Intracellular ROS generation after exposure to the studied formulations was determined using Cellular ROS/Superoxide Detection Assay Kit (ab113851, Abcam, Cambridge, United Kingdom). The cells were cultured in clear-bottom 96-well microplates, each at a density of 10^4^ cells/well, and allowed to adhere overnight in a 5% CO_2_ incubator at 37 C^o^. Then, the cultured cells were exposed to the combined formulation, Oxa and TPO-NANO, each at a concentration equivalent to its IC_50_, for 24 h. Then, the cells were stained with 2′,7′-dichlorodihydrofluorescein diacetate (DCFH-DA) using kits in line with the manufacturer’s instructions. The intracellular fluorescence intensity was quantified with a multi microplate reader at excitation and emission (Ex/Em) wavelengths of 485 and 535 nm, respectively, and it was expressed as a fold change in fluorescence intensity relative to untreated control.

### 2.12. Assessment of apoptosis/necrosis using flow cytometry

The effect of TPO-NANO, Oxa-NS and their combination on programmed and non-programmed cell death, and the amount and location of phosphatidylserine in normal, apoptotic, and necrotic HCT116 and HT-29 cells, were evaluated using annexin V-FITC/propidium iodide detection Kit (ab14085) according to the manufacturer’s instructions. The cancer cells were cultured in 6-well plates, each at a density of 1 × 10^6^ cells/well in 2 ml of medium. Apoptosis and necrosis cells were quantified after treatment with combined formulation and the individual drugs at doses equivalent to their respective IC_50_ values, in a 5-% CO_2_ incubator for 24 h at 37 °C. The cells were subsequently trypsinized, washed twice with ice-cold buffer, resuspended in 1X binding buffer (500 µl), and stained with annexin V-FITC (5 µl) and propidium iodide (PI, 5 µl) for 15 min in the dark at room temperature. Then, the cells were injected into ACEA Novocyte™ flow cytometer (ACEA Biosciences Inc., San Diego, CA, USA) and the analyses were carried out at extinction/emission wavelengths of 488/530 nm bandpass for FITC staining, and at extinction/emission wavelengths of 535/617 nm for PI staining. Annexin V-positive cells were apoptotic; cells positive for PI staining were only counted as early apoptotic, while cells + ve for PI and annexin V were at the late apoptotic stage. The flow cytometric data were measured with quadrant analysis and calculated using ACEA NovoExpress™ software (ACEA Biosciences Inc., San Diego, CA, USA).

### 2.13. Determination of protein levels of p53 and bax in HCT116 and HT-19 cells

The protein levels of p53 and Bax in cell supernatants were measured using commercial ELISA kits (Abcam, Cambridge, UK), based on the manufacturer’s protocols.

### 2.14. Colony formation assay

The effects of the studied formulations on cancer cell proliferation were determined with clonogenic assay. The HCT116 and HT-29 cells were cultured in 6 well-plates for 24 h, each at a density of 500 cells per well in 2 ml of medium (control) or 2 ml of medium containing combined formulation or individual drugs, each at a concentration equivalent to its IC_50_. After the treatments, the cells were washed twice with PBS and kept in a 5% CO_2_ incubator at 37 C^o^ for 7 days, during which the medium was changed every 2 days. For fixation and staining, the medium was removed, and the cells were washed with PBS and fixed with 0.5% crystal violet for 30 min at room temperature. Then, the plates were washed with water and air-dried. The number of colonies containing more than 50 cells was determined under a light microscope. Cell survival fraction (SF) and plating efficiency (PE) were calculated as follows:

PE=(colony number in treated well ÷ initial number of seeded cells)*100

SF=(colony number in treated well ÷initial number of seeded cells)*PE

### 2.15. Statistical analysis

The experimental runs were formulated according to the Box-Behnken design (BBD) of response and surface methodology analysis using MINITAB^®^ software (Minitab Inc., USA). Statistical analysis was performed with MegaStat version 10.3. Butler was applied to determine significant differences among the groups. Each experiment was performed in triplicate, and the values are expressed as mean ± SD. Differences were considered statistically significant at *p* ≥ 0.05.

## 3. Results and discussion

### 3.1. Optimization of TPO-NANO by Box-Behnken design

In the present investigation, Box-Behnken design (BBD) was applied to analyze the impacts of three input variables: % T80, % TPO and ratio of PG: T80 on out variables, i.e., droplet diameter and PDI. As summarized in Table S1, fifteen batches were generated using varying proportions of the three input variables, and their effects on the response variables were determined. The experimental findings showed that the droplet diameter was between 12.76 ± 0.04 to 367 ± 14.14 nm, and PDI varied from 0.04 ± 0.01 to 0.16 ± 0.08. These data reveal the homogenous size distribution of the prepared TPO-NANO formulations (Bi et al., 2021). Based on BBD, the measurable effects of individual and combined input variables (A, B and C) resulted in different output variables for droplet diameters and PDI. Each response variable was separately fitted to a polynomial equation as follows:

(Eq1) Droplet diameter = −3746 + 718.0 A + 758 B + 18888 C − 37.86 A*A + 683.2 B*B − 50795 C*C − 89.2 A*B − 442.0 A*C − 8556 B*C 

(Eq2) PDI= 0.214 + 0.0613 A − 1.300 B + 3.199 C − 0.00775 A*A + 0.543 B*B − 2.04 C*C + 0.0875 A*B− 0.1406 A*C − 2.381 B*C

**Figure 1.**
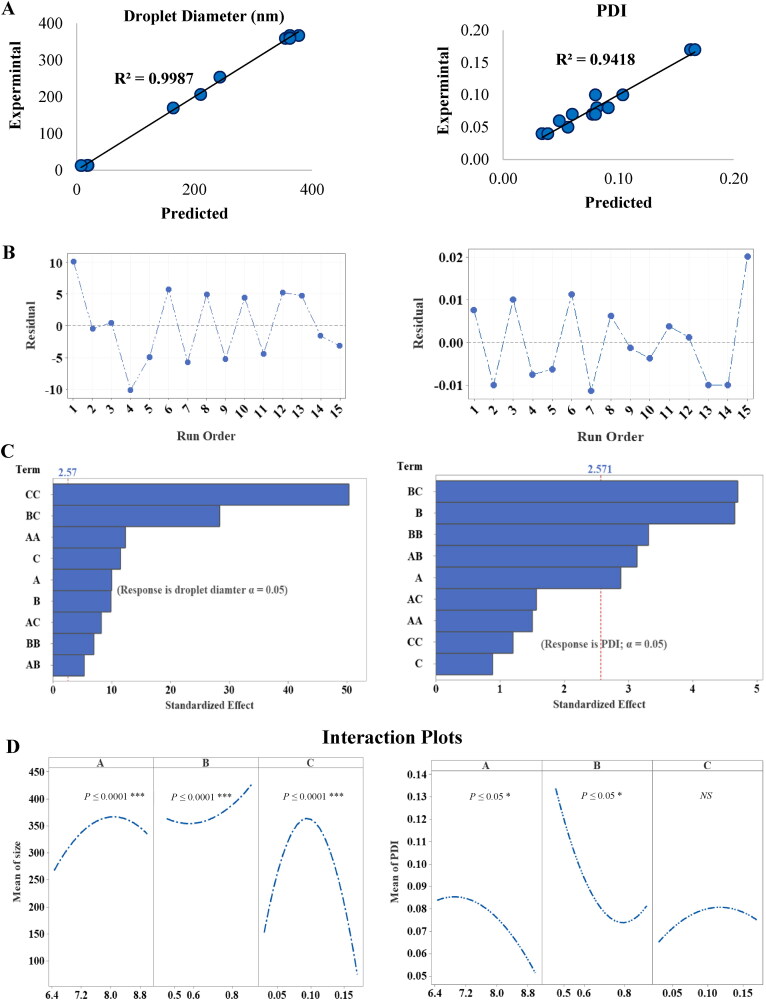
(A) Linear correlation plots of predicted vs experimental values for Size and PDI, and (B) the corresponding residual plots. (C) Pareto Chart of standardized effects of the input variables. (D) Main effects plot from BBD for droplet diameter and PDI.

These equations describe the effect of input factors (A, B and C) and their interactive terms on output variables. The suitability of BBD and the significance of models were assessed with ANOVA test, lack of fit test, and multiple correlation coefficient (R^2^) test (Table S2). The lack of fit *p*-value was > 0.05, relative to pure error, indicating very small variations in data around the fitted values for response variables. In addition, the predicted values of droplet diameter and PDI from the equations (Eq_1_ and Eq_2_, respectively), were quantitively compared with the experimental values, and their corresponding residual plots are shown in Figure 1(A) and (B). The high R^2^ and R^2^-adj values of response variables close to 1 for droplet diameter and PDI, demonstrated the closeness of data to the mean, and indicated a good statistical model. The predicted coefficient (R^2^-Pred) values for droplet size and PDI were 98.12 and 74.50%, respectively, indicating the predictability and validity of the generated models. The effectiveness of an input variable is deemed to be significant when the effect of a factor is not equal to zero and *p* > 0.05. The opposite effect is assigned a negative sign, whereas a positive sign is assigned to agonist effects of input variables on response.

#### 3.1.1. Effects of input variables on droplet diameter and polydispersity index

The results showed that droplet size varied from 12.76 ± 0.04 to 367 ± 14.14 nm. In the polynomial equations (1 and 2), the model F-values were high. Therefore, it may be inferred that the models were significant. As displayed in Figure 1(C), Pareto charts of droplet diameter showed that all linear, interactive, and quadratic terms were effective (*p* ≤ 0.05), whereas in terms of PDI, the largest effect was caused by the following interactive terms: B*C, B, B*B, A*B and A which were the most effective (*p* ≤ 0.05). Figure 1(D) shows the main effects plots of input variables on droplet diameter and PDI. High percentages of T80 and TPO led to the coalescence of nanodroplets which enhanced increase in average size, while increases in PG: T80 ratio led to significant reductions in droplet size (*p* ≤ 0.05). Regarding PDI, it was observed that increasing the proportions of T80 and TPO caused corresponding decreases in PDI (*p* ≤ 0.05), and it showed a non-linear relationship, while the effect of PG: T80 was not significant (*p* < 0.05).

**Figure 2.**
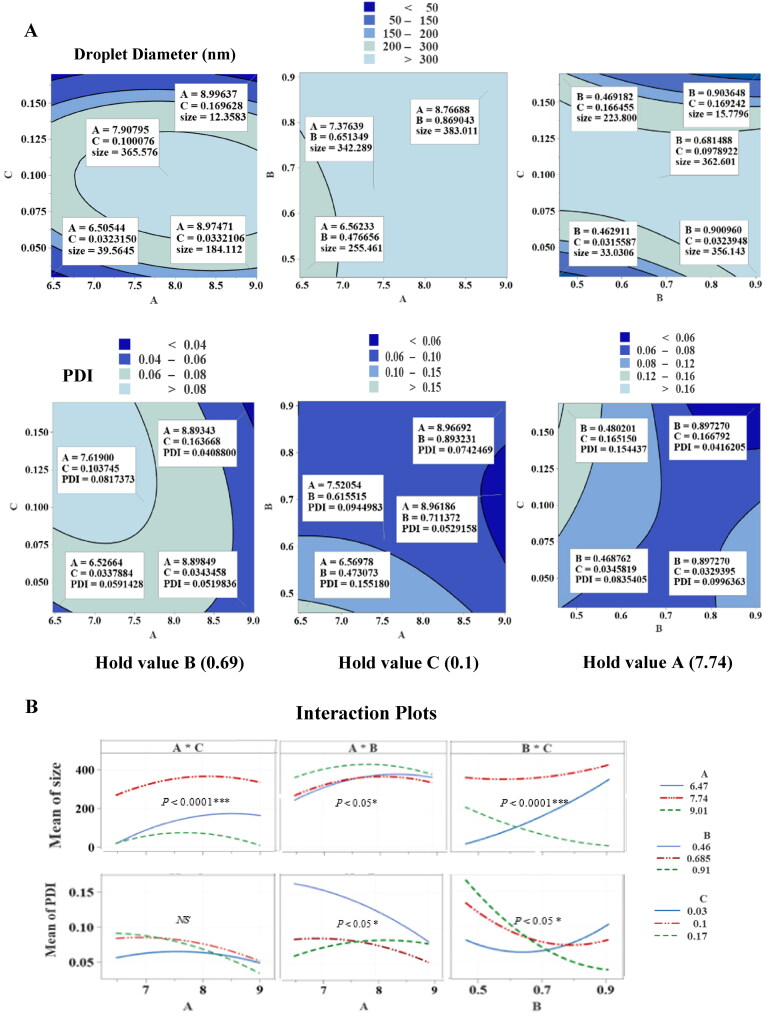
Graphic representation of the effects of input variables [% T80 (A), % TPO (B), and PG: T80 ratio (C)] on droplet diameter and PDI. (A) Contour plots. (B) Interaction plots of prepared TPO-NANO showing hold values.

The contour and interaction plots of output variables are presented in Figure 2(A) and (B). Figure S3 shows response surface plots as functions of A*C, B*C and A*B, indicating the impact of input factors when the third factor was kept at a fixed median level. In the A*C contour plot, increasing the concentration of T80 and PG resulted in a marked reduction in droplet diameter from 39.56 to 12.36 nm (*p* ≤ 0.05). This correlation was clear when % T80 and PG: T80 ratio increased to 9 and 0.17%, respectively at the mid-level of TPO (0.68%), while no statistically significant change was observed in PDI value, and it appeared with an absence of curvature in the 3-dimensional surface response plot (*p* < 0.05). However, when 7.9% T80 was combined with PG: T80 of 0.1 at the mid-value of TPO, the maximum droplet diameter observed was 365.58 nm. Regarding the A*B interaction term, the droplet size of TPO-NANO was significantly increased from 255.46 to 383.01 nm, while the PDI value was decreased by 50% when 9% T80 was combined with 0.91% TPO at the mid-level of PG: T80 (*p* ≤ 0.05).

**Figure 3.**
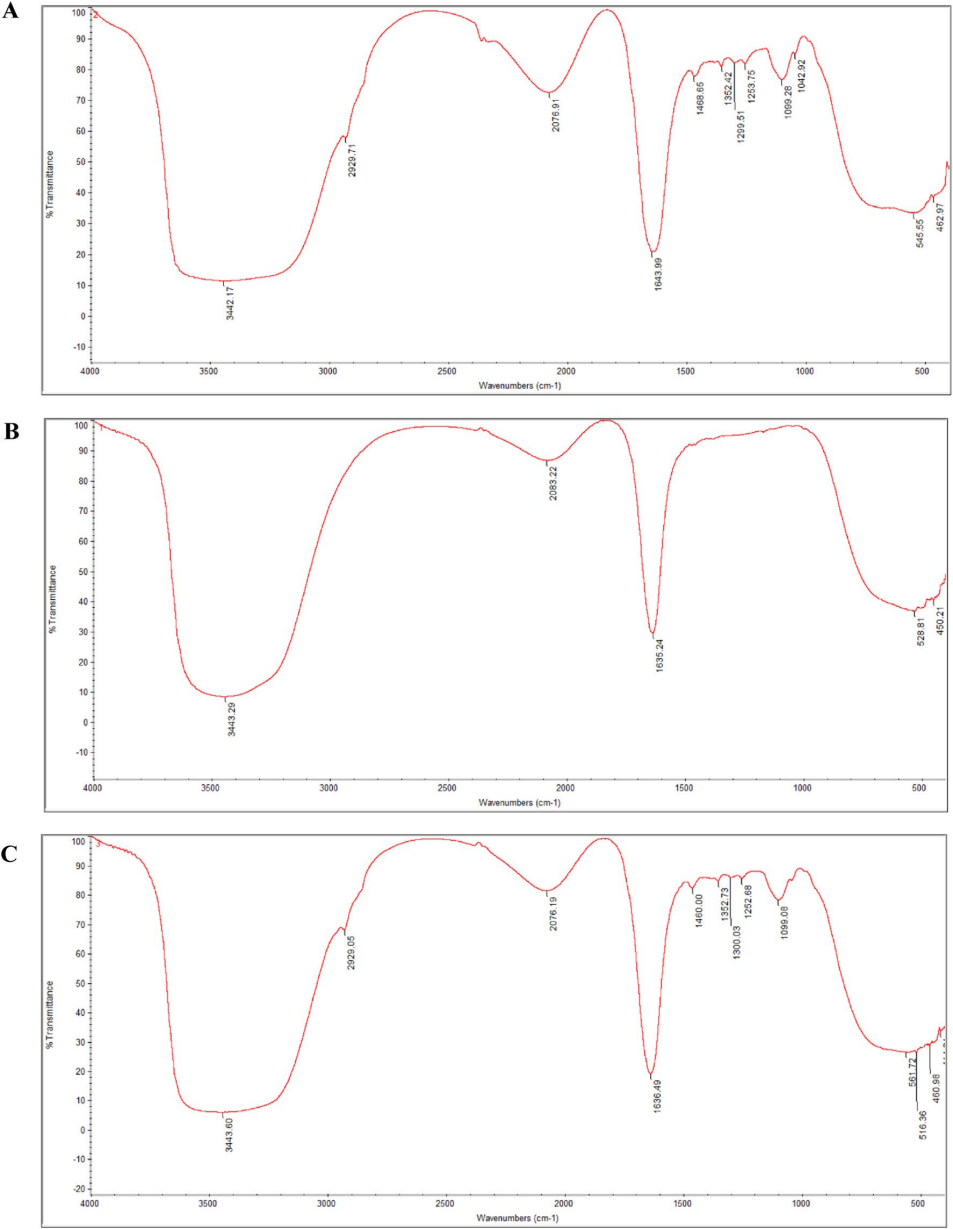
FTIR spectrum of TPO-NANO (A), Oxa-NS (B) and Oxa + TPO-NANO (C).

The contour plots for droplet diameter and PDI for the most effective interaction term, B*C, at the mid-level of T80 (7.74%) revealed a marked reduction in the droplet diameter from 33.03 to 15.78 nm, and a decrease in PDI from 0.08 to 0.04 when the maximum ratio of PG: T80 (0.17%) interacted with a maximum percentage of TPO (0.91%). The presence of curvature in the surface response curve of B*C indicated that the interaction term had a significant effect on particle diameter and PDI of the prepared nano-formulations (*p* ≥ 0.05). It is likely that the surfactant molecules reached an optimum concentration and achieved maximum coverage, and rapidly adsorbed onto the surface of the nano-droplets, therapy reducing the interfacial tension at the oil-water interface, resulting in the formation of smaller droplets (Qian & McClements, 2011; Witayaudom & Klinkesorn, 2017; Aziz et al., 2013; Joung et al., 2016; Manchun et al., 2014). In addition, the presence of maximum PG: T80 ratio (0.17%) as a co-surfactant worked in combination with T80 to enhance the flexibility of the interfacial film by positioning itself between the T80 tails; this reduced clustering and promoted the production of a homogenous size of oil nanodroplets in the aqueous phase (Polychniatou & Tzia, 2014). On the other hand, at the mid-level of T80, increasing %TPO at low and fixed PG: T80 caused a sharp increase in droplet diameter (*p* ≤ 0.05). This observation is in agreement with a previous finding Troncoso, Aguilera (Troncoso et al., 2012) which indicated that increases in droplet diameter as oil concentration increased in the liquid phase might be due to increases in the viscosity of the oil. The presence of a high volume of oil enhanced a change from turbulent flow to laminar flow during the formation of oil nanodroplets using the high-pressure homogenization technique. This resulted in reduced efficiency and decreased rate of disruption of droplets to smaller sizes. On the other hand, the presence of undissolved droplets of TPO-NANO as a result of increased oil concentration at a low and fixed PG most likely reduced the interfacial tension at the interface between the two phases. This resulted in coalescence and enhanced flocculation in water, leading to aggregates of droplets due to their hydrophobic properties, and hence larger droplet size and PDI values (Niemann & Sundmacher, 2010; Tadros, 2013; Uluata et al., 2016; Maindarkar et al., 2013; Rao & McClements, 2012; Choudhury et al., 2019; Chuesiang et al., 2018; Cheong et al., 2018; Sharma et al., 2016; Musa et al., 2013; Guttoff et al., 2015; Komaiko & McClements, 2014). At low % TPO and PG: T80 ratio, the amount of non-adsorbed micelles of T80 exceeded the critical level, making them aggregate around TPO droplets, resulting in a thick-density layer which probably tended to induce a birding effect that increased the size and PDI of the nanodroplets (Uluata et al., 2016; Liu & Wang, 2014; Wang et al., 2009; Kim et al., 2014).

#### 3.1.2. Optimization conditions for preparing TPO-NANO formulation

The optimal conditions for producing TPO nano-emulsions used in this study resulted in a stable formula. The optimum TPO nano-emulsion formula was expected to have minimum values of droplet size and PDI. The results showed that the optimal formulation had droplet diameter and PDI of 12.90 ± 0.04 nm and 0.04 ± 0.009, respectively. It was composed of 7.5 vol % of T80, 0.17 vol % of PG: T80 (HLB =13.14) and 0.90% TPO in the aqueous phase. Table S3 reveals that the droplet size and size distribution resulting from the optimized conditions were in good agreement with the predicted values (*p* < 0.05). This confirms the validity and predictability of generated models.

### 3.2. Physical characterizations of loaded nano-formulation (oxa + TPO-NANO)

To generate the loaded formulation, Oxa was incorporated into the selected nano-formulation at a concentration of 100 µM. In addition, 100 µM of free drug solution (Oxa) was prepared by dissolving 40 µg of the drug in 1000 µl of physiological saline [0.90% (w/v) NaCl]. Results obtained from measurements of droplet diameter and PDI are presented in Figure S4. There was significant enlargement of droplet size from 12.90 ± 0.04 to 14.47 ± 0.53 nm (*p* ≤ 0.05). However, the PDI values were not significantly changed when Oxa was added to the formulation (*p* < 0.05). This is considered an indicator of the drug loading inside the droplets (Al-otaibi, 2021; AlMotwaa et al., 2020).

**Figure 4.**
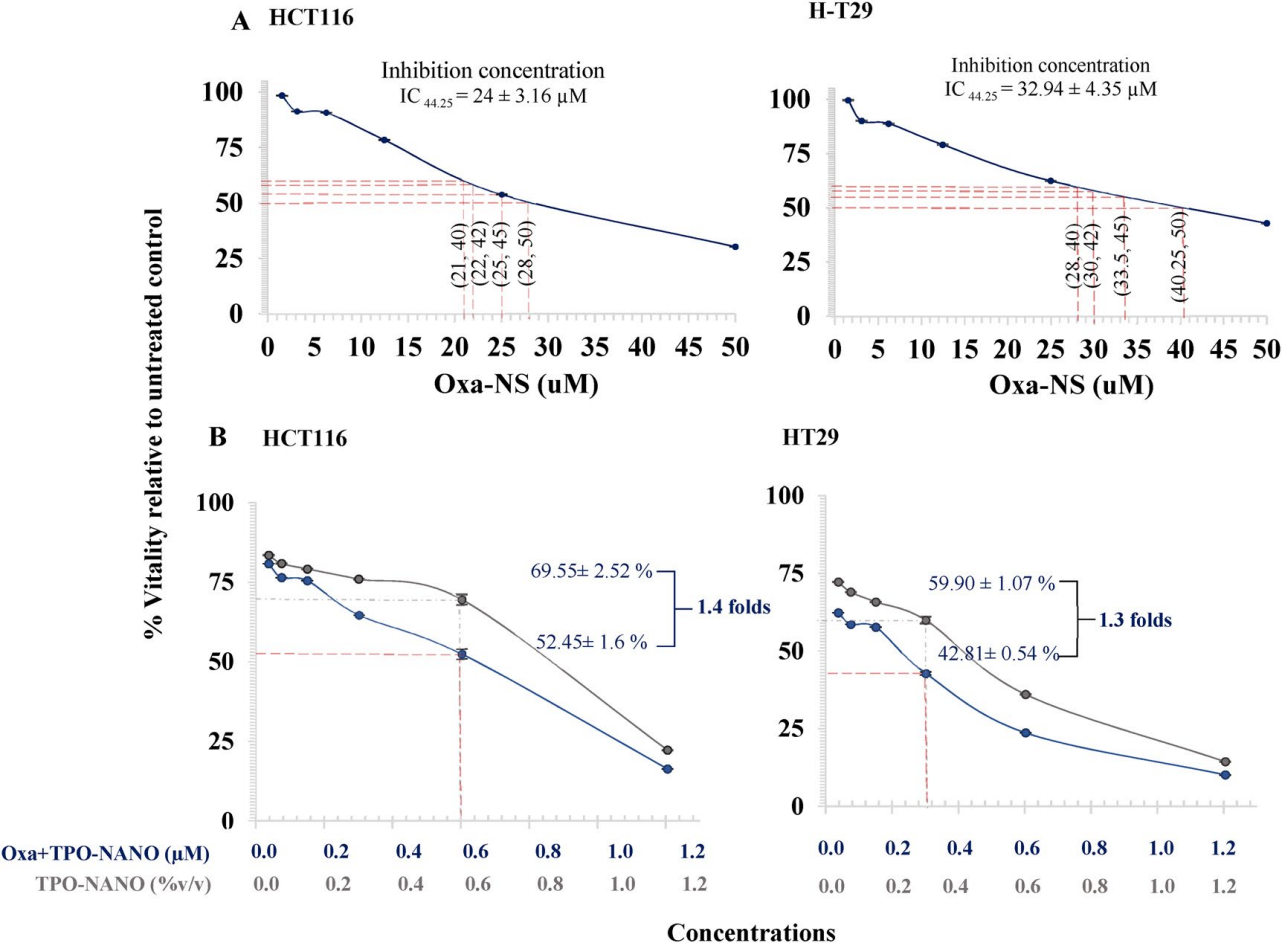
Dose-dependent effects of Oxa-NS (A), TPO-NANO (B) and Oxa + TPO-NANO (C) on viability of HCT116 and HT-29 colon cancer cell lines, relative to untreated control. ***P < 0.001, compared with the untreated group. All data are presented as mean ± SEM of three independent experiments.

### 3.3. FTIR characterization

The FTIR spectra of the studied formulations obtained in the range of 1000 to 4000 cm ^−1^ for analysis of the interaction between TPO-NANO and Oxa are presented in Figure 3(A) and (B). In the FTIR spectra, absorption of Oxa-NS occurred at 3443 and 1635 cm^−1^, which were due to the *v* (OH) (Moghaddasi et al., 2018; Yilmaz et al., 2019) and C = O stretching vibrations (Yang et al., 2018; Yasmeen et al., 2016). Regarding Oxa + TPO-NANO spectrum, FTIR absorption bands were seen at 3442, 2929, 2076, 1643, 1468, 1352, 1299, 1253, 1099 and 1042 cm^−1^, due to OH stretching vibration (Moghaddasi et al., 2018; Yilmaz et al., 2019), asymmetric CH_2_ stretching vibration (Yilmaz et al., 2019; Ravindran et al., 2013), C = O stretching vibration (Yang et al., 2018; Yasmeen et al., 2016), C-H binding vibration in the range of 1468 to 1352 cm^−1^ (Panwar et al., 2015; Farber et al., 2019; Choowang et al., 2018), and C-O stretching vibration in the range of 1299 and 1042 (Yang et al., 2018; Choowang et al., 2018; Vahur et al., 2011; Ibrahim et al., 2011). The spectrum showed most of the peaks from TPO-NANO. They were slightly shifted, but no changes or additional peaks were detected in Oxa + TPO-NANO, thereby confirming the molecular/structural integrity and stability of the nano-emulsion formulation (Moghaddasi et al., 2018). The resemblance between the spectra shown in Figure 3(A) and (C) confirms the presence of the drug in the loaded nano-emulsion (Chouhan & Bajpai, 2009).

### 3.4. Evaluation of the anticancer activity of effect of Oxa-NS, TPO-NANO, and their combination

#### 3.4.1. In vitro cell viability and alteration in cell morphology

The toxic effects of the free drug, Oxa-NS, and TPO-NANO and the drug combination on HCT116 and HT-29 cells were determined after 24-h exposure, and the values of percentage viability were estimated. As shown in Figure 4, HT-29 showed 55.75 ± 4.35% inhibition on exposure to 32.94 ± 4.35 µM Oxa-NS, which was 1.4 folds higher than the level needed for 55.75 ± 4.35% inhibition of HCT116 cells (24 ± 3.16 µM, *p* ≥ 0.05). It was previously demonstrated that Oxa had a high IC_50_ for the p53-mutated HT-29 cell line, relative to the wild-type HCT116 cell line (Toscano et al., 2007). Furthermore, the cytotoxic effect of TPO-NANO on HCT116 and HT-29 cell lines was investigated before and after the incorporation of Oxa into the formulation. The HCT116 cells had 50.33 ± 2.52% inhibition after treatment with 0.86 ± 0.05% (v/v) TPO-NANO, while HT-29 cells appeared more susceptible to the formulation, with 50.33 ± 2.52% growth inhibition at half the volume % of the TPO-NANO needed for 50% inhibition of HCT116 cells [0.42 ± 0.54%(v/v), *p* ≥ 0.05].

**Figure 5.**
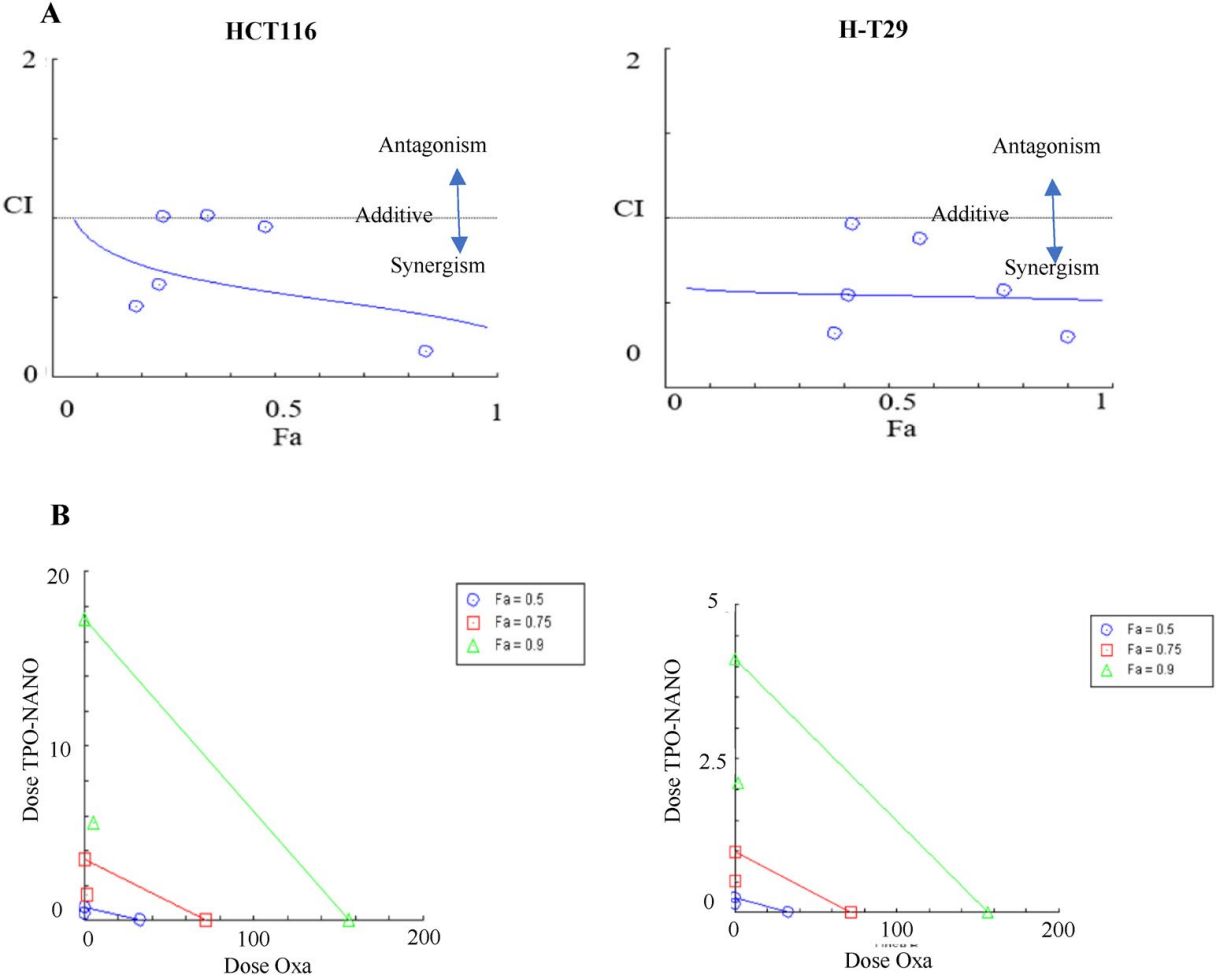
Graphic representations obtained from the CompuSyn report for effects of Oxa-NS and TPO-NANO and their combinations on HCT116 and HT-29 cell lines. (A) Fa-CI plot (Chou-plot) showing synergistic interaction between Oxa-NS and TPO-NANO, and (B) Isobolograms.

To determine which combination ratio reduced cell vitality due to synergistic effect, the Compusyn software was used to determine combination index (CI) values, and the isobologram plots which were obtained at 0.5, 0.75 and 0.9 of fraction affected as shown in Figure 5 to quantitatively evaluate the antagonism and synergistic interactions between the two drugs. The combined formulation had a greater cytotoxic effect on the two cell lines than either TPO-NANO or Oxa when utilized alone. Regarding HCT116, a synergism between the two drugs was observed, with a fraction affected (Fa) of about 0.48, and a CI of 0.94 at concentrations of Oxa + TPO-NANO (0.6% (v/v) TPO-NANO + 0.6 µM Oxa) and the viability of HCT116 cells reduced 1.4-folds as compared with HCT116 cells treated with 0.6%(v/v) TPO- NANO), *p* > 0.05. On the other hand, the combined formulation caused a 40-fold reduction in the dose of Oxa needed for 50% growth inhibition, when compared with HCT116 cells treated with Oxa-NS (*p* ≥ 0.05). The Fa-CI plot for HT-29 cells showed that Oxa + TPO-NANO exhibited a synergistic effect, with fractional inhibition of 0.57 and a CI value of 0.88 when 0.3 µM Oxa was combined with 0.3% (v/v) TPO-NANO, and cell viability was reduced 1.3-fold, when compared with HT-29 cells treated with 0.3% (v/v) TPO-NANO. When compared with Oxa-NS treated group, the combined formulation caused a 109.8-fold reduction in the dose of Oxa needed for 50% growth inhibition, relative to HT-29 cells treated Oxa-NS (*p* ≥ 0.05). This observation could be attributed to the enhancement of the penetration of chemotherapeutics through the cell membrane when delivered into nano-formulations, in addition to the cytotoxic potential of essential oils on cancer (Al-otaibi, 2021; Zhang et al., 2019; AlMotwaa, 2021; Al-Otaibi et al., 2020).

**Figure 6.**
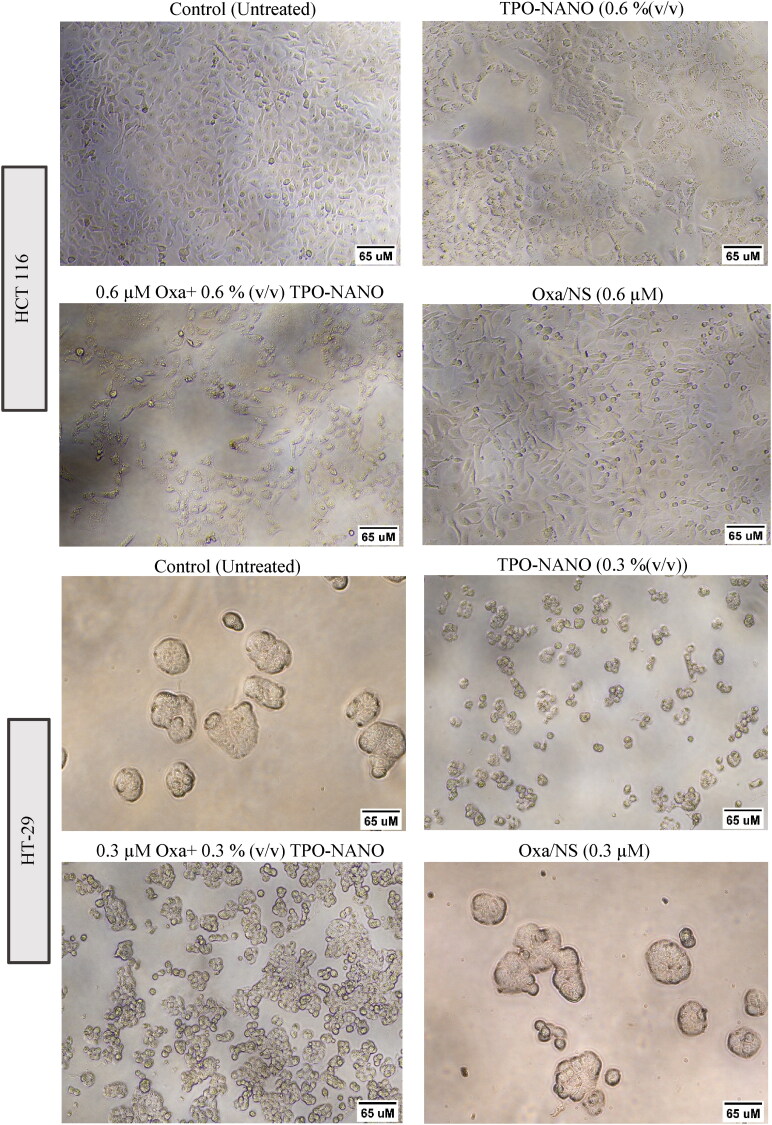
Morphological alterations of HCT 116 and H-T29 cell lines after treatment for 24 h with selected concentrations of the studied formulations. The images were taken under an Olympus inverted light microscope at a magnification of x200.

These inhibitions in growth rates were accompanied by significant degrees of apoptotic cell death which were confirmed by characteristic morphological alterations in HT-29 and HCT116 cells seen under an inverted microscope (Figure 6). The untreated cells were homogeneously distributed on a culture field, with HCT116 exhibiting polygonal-shaped colonies, while HT-29 cells showed an elliptical shape of colonies. Following incubation with Oxa + TPO-NANO [0.6 µM + 0.6% (v/v)] and TPO-NANO [0.6% (v/v)], there was significant growth inhibition in HCT116 cells, in addition to much-reduced confluency and transformation of the shapes of the cells from polygonal to circular, resulting in cell shrinkage, relative to cells treated with 0.6 µM Oxa-NS. In HT-29 cells, the most obvious morphological changes were caused by treatment with Oxa + TPO-NANO [0.3 µM + 0.3% (v/v)] or with TPO-NANO [0.3% (v/v)], and the cells appeared smaller, rounded, and contracted, with cellular blebbing and damaging interaction with neighboring cells, and they were more significantly destroyed than cells treated with 0.3 µM Oxa-NS. The cells became shrunken, with apoptotic body formation. Therefore, subsequent experiments were performed based on these selected ratios since they produced synergistic effects against HCT116 and HT-29 cancer cell lines at the half-maximal inhibitory concentration (IC_50_) of the combined formulation.

#### 3.4.2. Effect of Oxa-NS, TPO-NANO, and their combination on apoptosis/necrosis of Colon cancer cells

**Figure 7.**
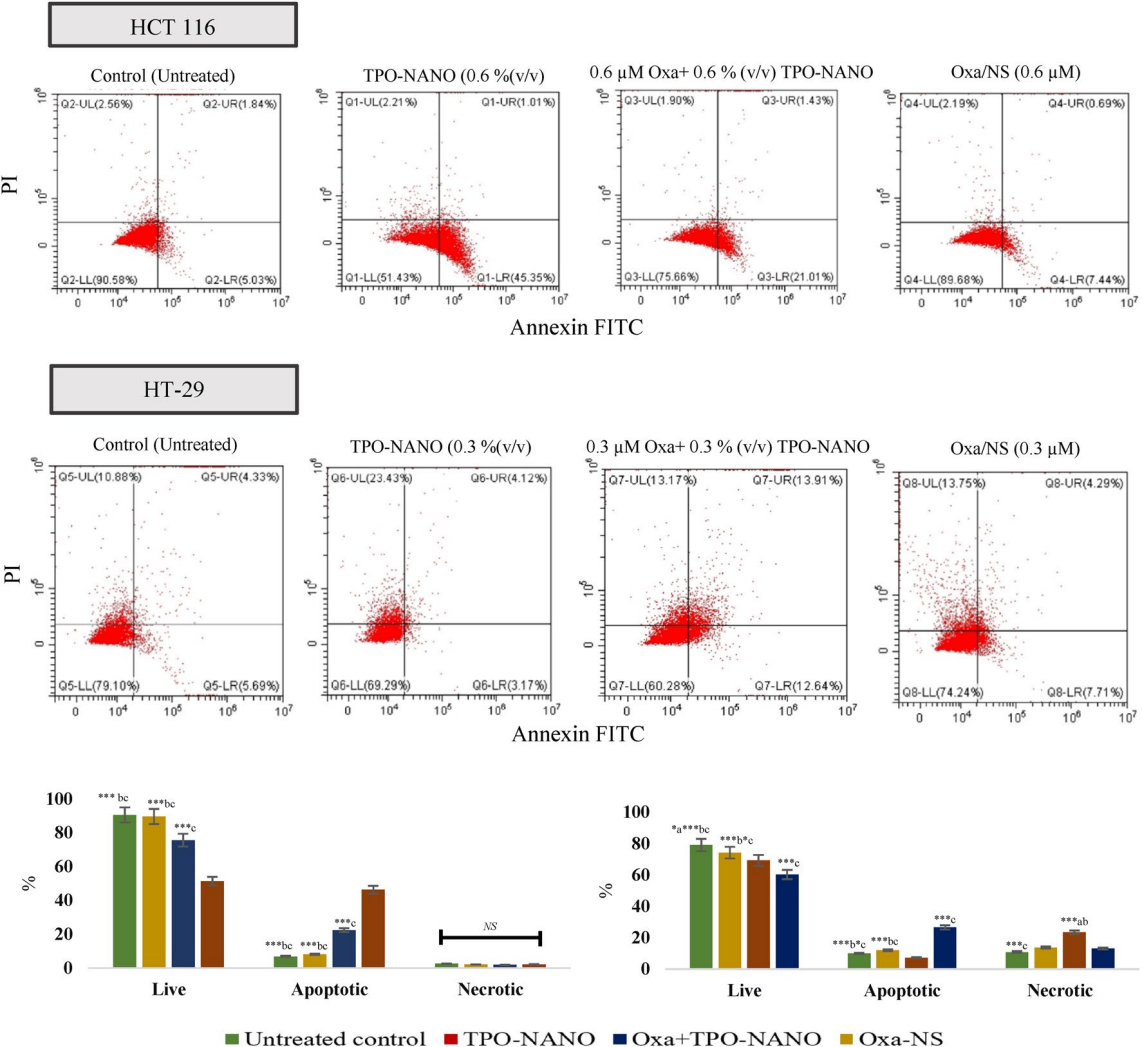
Apoptotic and necrotic assessment of TPO-NANO, Oxa-NS and Oxa + TPO-NANO in HCT 116 and H-T29 cell lines. The cells were exposed to the samples for 24 h and stained with Annexin-V/FITC and propidium iodide. (B) Percentages of cell death, as revealed from flow cytometric analysis after annexin V staining. Values are expressed as mean ± SD (*n* = 3). *Significant differences amongst the groups (*p ≤ 0.05, **p ≤ 0.001, ***p ≤ 0.0001; ^a, b, c^ represents Oxa-NS, Oxa + TPO-NANO, and TPO-NANO, respectively. *^NS^* Indicates not statistically significant.

To investigate the mechanism of cell death induced by TPO-NANO, Oxa-NS, and their combination, HCT116 and HT-29 cell lines were stained with annexin-V/FITC and PI and analyzed with flow cytometry after exposure to the predetermined IC_50_ of the combined formulation (Oxa + TPO-NANO), and to the individual agent. As shown in Figure 7, HCT116 cells treated with 0.6% (v/v) TPO-NANO had the highest proportion of apoptosis among the groups (*p* ≥ 0.05), followed by cells treated with Oxa + TPO-NANO [0.6% (v/v) TPO-NANO + 0.6 µM Oxa] which exhibited 2.8- and 3.3-folds increase when compared with cells treated with 0.6 µM Oxa-NS and control, respectively (*p* ≥ 0.05). Regarding HT-29 cell lines, a 2.3-fold increase in necrotic cell death was observed when treated with 0.3% (v/v) TPO-NANO, relative to untreated cells (*p* ≥ 0.05). When HT-29 cells were treated with 0.3 µM Oxa combined with 0.3% (v/v) TPO-NANO, 2.6- and 3.6-fold increases in proportions of apoptotic cells were observed, when compared to the control and TPO-NANO groups, respectively, and there were increased proportions of cells in the early and late phases of apoptosis (*p* ≥ 0.05). On the other hand, the percentage of apoptosis in HT-29 cells treated with 0.3 µM Oxa-NS was significantly lowered, when compared with cells treated with loaded delivery system (*p* ≥ 0.05). These results could be due to increased accumulation of drugs and enhanced penetration of nanocarrier which might have modulated the expressions of numerous cellular factors associated with the cellular apoptotic pathway, resulting in enhanced cytotoxic effects against cancer cells (Farhoudi et al., 2022). This explanation is consistent with the following experimental findings:

#### 3.4.3. Oxa + TPO-NANO induced apoptosis via ROS-mediated apoptosis in HCT116 and HT-29 cancer cells

**Figure 8.**
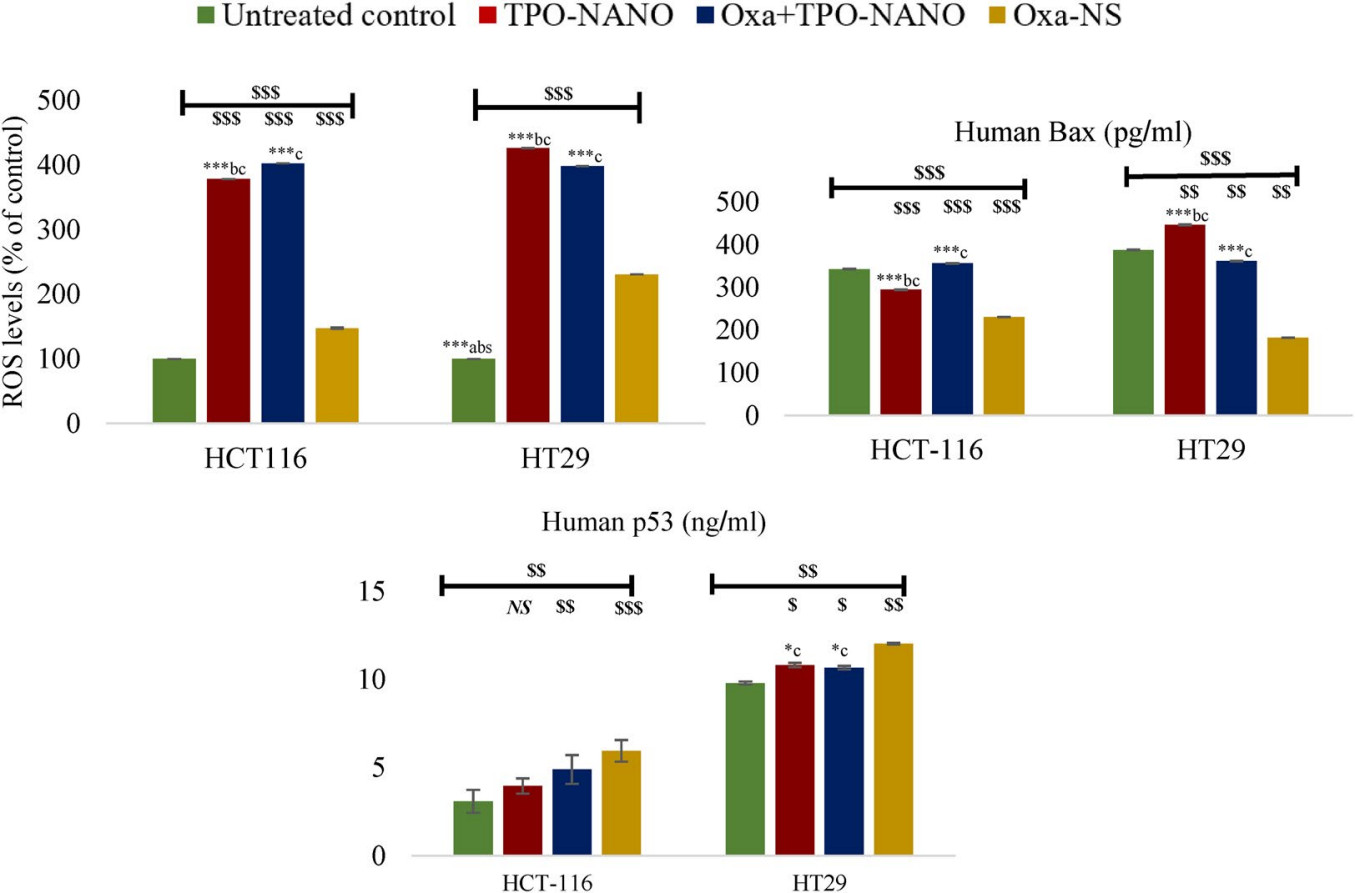
ROS concentration in HCT 116 cells after 24 h of exposure to 0.6 % (v/v) TPO-NANO, 0.6 µM Oxa-NS, and their combination (Oxa + TPO-NANO); and ROS levels in HT-29 cancer cell lines exposed for 24 h to 0.3 % (v/v) TPO-Nano, 0.3 µM Oxa-NS, and their combination (Oxa + TPO-NANO). The percentage change in fluorescence intensity of treated cells was quantified relative to untreated control. Data are expressed as mean ± SD; *n* = 3. ^$^*P* ≤ 0.05, ^$$^*p* ≤ 0.001, ^$$$^*p* ≤ 0.0001, versus control; *^NS^p* < 0.05, versus control. *Significant differences amongst the treated groups (*p ≤ 0.05, **p ≤ 0.001, ***p ≤ 0.0001; ^a, b, c^ represent Oxa-NS, TPO-NANO and Oxa + TPO-NANO, respectively.

To determine whether Oxa + TPO-NANO stimulated ROS-mediated apoptosis, DCFDA assay was employed to evaluate intracellular ROS levels after treating the cells with the formulations for 24 h. The results are presented in Figure 8. Compared to the untreated group, ROS levels in HCT116 cells were significantly elevated 4-fold and 3.8-fold after treatment with Oxa + TPO-NANO and TPO-NANO, respectively, whereas Oxa-NS induced only a slight (0.5-fold) increase in ROS levels, relative to the control **(***p* ≥ 0.05). Treatment of HT-29 cells with TPO-NANO resulted in a high elevation in ROS levels, followed by Oxa + TPO-NANO, when compared with Oxa-NS, with just a 1.3-fold increase in ROS, relative to the control **(***p* ≥ 0.05). The marked elevations in ROS levels in cells treated with Oxa + TPO-NANO and TPO-NANO, relative to cells treated with Oxa-NS might be explained by the apoptotic potential of these two formulations on the studied cancer cells. Previous studies have demonstrated the anticancer potential of essential oils formulated into nanocarriers which predominantly triggered ROS production within cancer cells, thereby facilitating apoptosis of the treated cells (Al-otaibi, 2021; AlMotwaa, 2021; Salehi et al., 2020; Panyajai et al., 2022; Nirmala et al., 2020). Furthermore, TPO and its bioactive components have been shown to exert anticancer potential predominantly by induction of ROS generation and induction of cell death through the mitochondria-mediated apoptotic pathway (Su et al., 2013; Bai & Tang, 2020; Li et al., 2013; Sobral et al., 2014; Pan et al., 2016; Karan et al., 2018).

#### 3.4.4. Levels of bax in treated HCT116 and HT-29 cancer cells

The elevated the levels of the proapoptotic protein Bax, in HCT116 and HT-29 cells treated with Oxa + TPO-NANO and TPO-NANO, relative to the Oxa-NS group, could be attributed to increased ROS generation which reduced the potential of the mitochondrial membrane, leading to mitochondrial dysfunction and increased release of Bax protein (Bhardwaj et al., 2016; Khan et al., 2018). This finding is consistent with an increase in the sensitivity of the cells to these formulations and their higher susceptibility to apoptosis, when compared to cells treated with Oxa-NS (*p* ≥ 0.05). The potential importance of Bax protein level in the sensitivity of cells to chemotherapeutics has been reported in previous works that correlated poor response to chemotherapy with decreased levels of Bax in cancer cells (Wang et al., 2019; Nazem et al., 2022; Khine et al., 2021).

#### 3.4.5. Levels of p53 in treated HCT116 and HT-29 cancer cells

The HCT116 cells had lower levels of p53 protein than HT-29 cells. This is due to the mutant p53 type in HT-29 cells which is characterized by overexpression of p53 protein more than the wild-type HCT116 (Toscano et al., 2007; Jang et al., 2019; Dabiri et al., 2019). However, treatment of HCT116 cells with Oxa-NS and Oxa + TPO-NANO resulted in marked increases in p53 levels relative to the control (*p* < 0.05), whereas cells treated with TPO-NANO exhibited p53 level similar to that of untreated cells (*p* < 0.05). Treatment of HT-29 cells with Oxa-NS produced the highest level of p53 protein, followed by cells treated with Oxa + TPO-NANO and TPO-NANO which had higher p53 levels than control cells (*p* ≥ 0.05). It was previously demonstrated that overexpression of mutated p53 with a reduced or absent function in cancer cells is often associated with drug resistance (Hientz et al., 2017). A study reported that mutant p53 HT-29 cells failed to activate p38 signaling, and had significantly less cytotoxicity and apoptosis than p53 wild-type HCT116 cell lines (Dabiri et al., 2019). However, some researchers have demonstrated that the inactive status of p53 is not a predictive factor for the apoptotic response of colon cancer cells to Oxa, suggesting that the function of p53 protein in the cellular response to Oxa may be due to the differences in their genetic profiles, and that other factors are likely to affect numerous pathways, thereby modulating cellular sensitivity to this drug (Arango et al., 2004; Seo et al., 2002; Petit et al., 2003).

#### 3.4.6. Clonogenic assay

**Figure 9.**
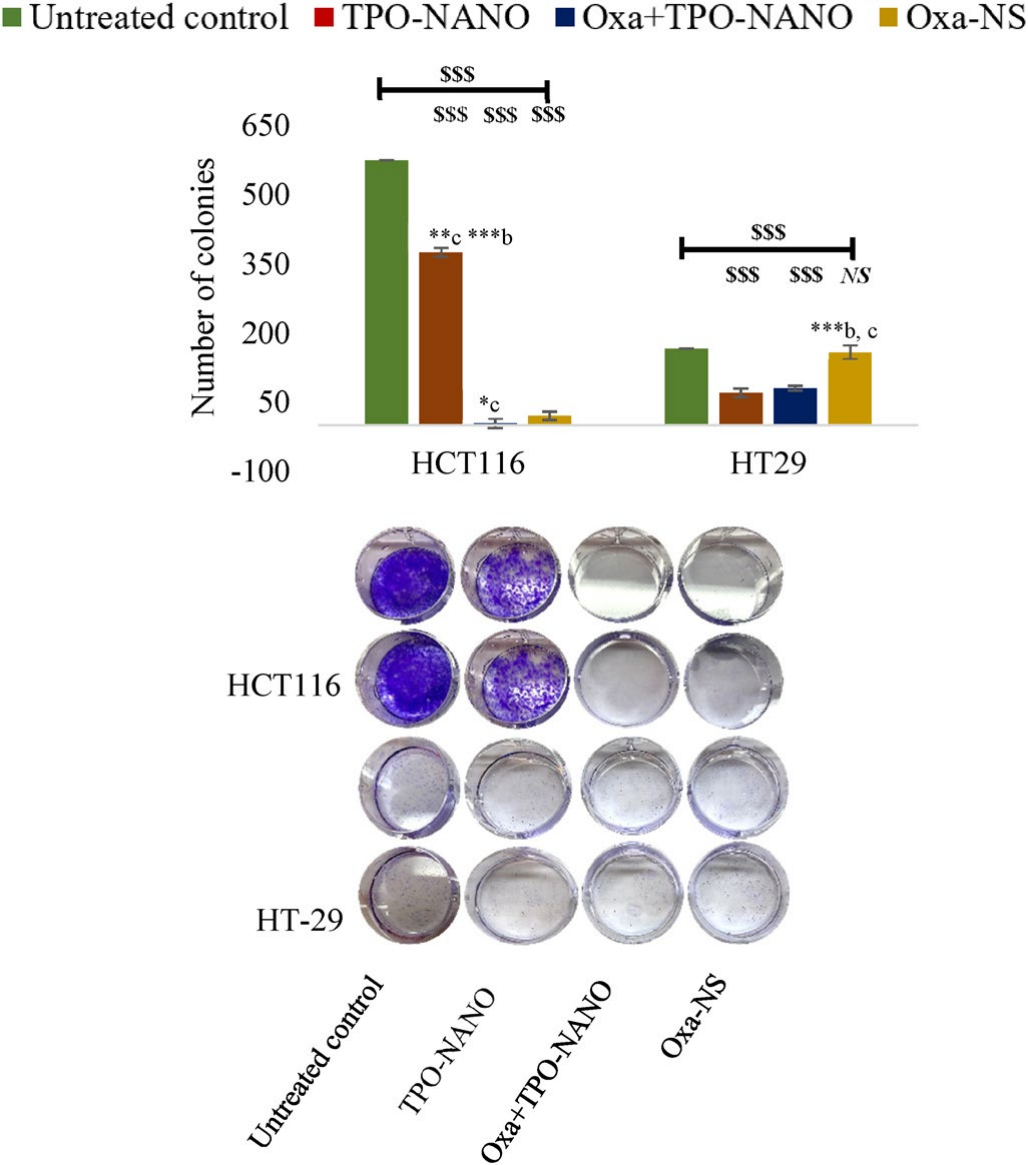
Inhibition of HCT116 cells after exposure to 0.6 % (v/v) TPO-NANO, 0.6 µM Oxa-NS, and their combination (Oxa + TPO-NANO), and inhibition of HT-29 colony growth after treatment with 0.3 %(v/v) TPO-Nano, 0.3 µM Oxa-NS, and their combination (Oxa + TPO-NANO) for 24 h. The colonies were allowed to grow for 7 days in normal media. The numbers of colonies were calculated relative to the untreated control, and the values are presented as mean ± SD (*n* = 3). ^$$$^*P* ≤ 0.0001, versus control; *^NS^p* < 0.05, no significant difference from control. *Indicates significant differences amongst the groups (*p ≤ 0.05, **p ≤ 0.001; ***p ≤ 0.0001). ^a, b, c^ represent TPO-NANO, Oxa + TPO-NANO and Oxa-NS, respectively.

To investigate the long-term implications of the reduced apoptosis and growth inhibition in response to the studied formulations, the survival of HCT116 and HT-29 cells was evaluated as a function of initial cell density for one week, before and after exposure for 24 h to individual drugs and Oxa + TPO-NANO at IC_50_. As shown in Table S4 and Figure 9, the percentage plating efficiency and survival fraction were significantly decreased after treatment with IC_50_ of Oxa + TPO-NANO in both cancer cells (*p* ≥ 0.05), indicating significantly higher suppression of potential for clonogenic formation in the cancer cells, when compared to treatment with individual drugs (*p* ≥ 0.05). The combined formulation (Oxa + TPO-NANO) comprising 0.6% (v/v) TPO-NANO + 0.6 µM Oxa almost completely annulled the ability of HCT116 cells to form colonies, when compared with 0.6 µM Oxa-NS alone **(***p* ≥ 0.05). Treatment of HT-29 cells with the combined formulation comprising 0.3% (v/v) TPO-NANO and 0.3 µM Oxa resulted in lower clonogenic potential than cells treated with 0.3 µM Oxa-NS alone (*p* ≥ 0.05). The higher reduction in colony formation in HCT116 cells after treatment with Oxa + TPO-NANO, relative to HT-29 cells, may be attributed to the combined effect of ROS-dependent Bax translocation and enhanced level of tumor suppressor p53 protein function in HCT116 cells, whereas HT-29 has a mutant-type p53 protein which enhanced the growth reduction independently via ROS-mediated to apoptosis of cancer cells (Arango et al., 2004; Dabiri et al., 2019; Kamran et al., 2022; Wu et al., 2019).

## 4. Conclusion

The present work reported the optimization of *Teucrium polium* L. essential oil nano-formulation (TPO-NANO with response surface methodology using Box-Behnken design to attain optimum formula in a short time with few experiments. This was done by selecting three independent factors: % Tween80 (% T80), % *Teucrium polium* L. essential oil (% TPO) and ratio of propylene glycol to tween 80 (PG:T80). The effect of TPO-NANO on the sensitivity of colon cancer cells with different p53 types: wild-type HCT116 and mutant-type HT-29 cells to Oxa treatment was investigated. Statistical analysis revealed significant impacts of the formulation parameters on the assessed response variables. The optimum terms selected [% T80 (7.7), % TPO (0.90) and PG: T80 ratio (0.17)] produced droplet diameter of 12.90 ± 0.04 nm and PDI of 0.04 ± 0.009. When Oxa was loaded to the optimized TPO-NANO, the droplet diameter was significantly increased to 14.47 ± 0.04 nm without any change in PDI value. The anticancer screening of Oxa + TPO-NANO demonstrated that it inhibited cellular growth and produced higher degree of morphological changes and apoptotic cell death in p53 wild-type HCT116 and p53 mutant-type HT-29 cancer cells, when compared to cells treated with Oxa-NS only. In addition, Oxa + TPO-NANO increased levels of ROS and Bax in the two cell lines and suppressed clonogenic formation more than individual treatments, regardless of p53 status. This is the first report showing that TPO-NANO enhanced the sensitivity of cancer cells to Oxa and triggered programmed cell death through ROS-mediated Bax translocation leading to mitochondrial apoptosis pathway in p53 wild-type and mutant type colon cancer cells. These findings suggest that the combined treatment strategy using Oxa + TPO-NANO might have potential therapeutic value for colon cancer patients. However, there is a need for verification and confirmation of these findings through more *in vitro* and *in vivo* studies (Kumar, 2019).

## Supplementary Material

Supplemental Material
