# Supplementary material for: Oxaliplatin-loaded nanoemulsion containing Teucrium polium L. essential oil induces apoptosis in Colon cancer cell lines through ROS-mediated pathway
Source: Drug Deliv. 2022 Jul 10;29(1):2190–205. doi: 10.1080/10717544.2022.2096711 (PMC9278420; doi:10.1080/10717544.2022.2096711)
Supplement: Supplemental Material [file IDRD_A_2096711_SM3736.docx]

**Supplementary materials**

**Oxaliplatin-loaded nanoemulsion containing Teucrium polium L. essential oil induces apoptosis in colon cancer cell lines through ROS-mediated pathway.**

Waad A. Al-Otaibi ^a ♣ *^and Sahar M. AlMotwaa ^a ♣^

^a^ Department of Chemistry, College of Science and Humanities, Shaqra University, Saudi Arabia

^♣^ These authors contributed equally

**Table of contents**

**Table S1.** **Comparative representation of levels of input factors and output variables from the fifteen experimental runs in Box-Behnken Design.**

**Table S2. Analysis of variance of the fitted linear, quadratic, and interactive models for output variables of TPO-NANO formulations.**

**Figure S3. Three-dimensional surface response plots showing the effects of input variables [% T80 (A) % TPO (B) and PG: T80 ratio (C)] on droplet diameter and PDI of prepared TPO-NANO showing hold-values.**

**Table S3. Comparison between predicted values and experimental values of droplet diameter and polydispersity index (PDI) of the optimized TPO-NANO.**

**Figure S4. Droplet diameter (nm) spectrum of TPO-NANO (A), and Oxa+TPO-NANO (B).**

**Table S4. Surviving fraction and plating efficiency of HCT116 and HT-29 cancer cell lines.**

**Table S1. Comparative representation of levels of input factors and output variables from the fifteen experimental runs in Box-Behnken Design.**

| Run Order | Input variables | | | Output variables | |
| --- | --- | --- | --- | --- | --- |
|  | **A** | **B** | **C** | **Droplet diameter (nm)** | **PDI** |
| 1 | -1 | -1 | 0 | 253.50± 45.25 | 0.17± 0.08 |
| 2 | +1 | -1 | 0 | 361.75± 6.01 | 0.065± 0.012 |
| 3 | -1 | +1 | 0 | 362.25± 5.2 | 0.063± 0.01 |
| 4 | +1 | +1 | 0 | 369± 19.24 | 0.074± 0.01 |
| 5 | -1 | 0 | -1 | 13.07± 0.53 | 0.06± 0.03 |
| 6 | +1 | 0 | -1 | 169.77± 6.52 | 0.1± 0.02 |
| 7 | -1 | 0 | +1 | 13.36± 0.90 | 0.067± 0.03 |
| 8 | +1 | 0 | +1 | 12.76± 0.03 | 0.04± 0.01 |
| 9 | 0 | -1 | -1 | 13.43± 0.95 | 0.07± 0.04 |
| 10 | 0 | +1 | -1 | 359.05± 43.77 | 0.16± 0.03 |
| 11 | 0 | -1 | +1 | 206.30± 61.23 | 0.05± 0.002 |
| 12 | 0 | +1 | +1 | 12.87± 0.05 | 0.05± 0.01 |
| 13 | 0 | 0 | 0 | 367± 14.14 | 0.069± 0.02 |
| 14 | 0 | 0 | 0 | 365± 10.24 | 0.07± 0.02 |
| 15 | 0 | 0 | 0 | 359.05± 43.77 | 0.1± 0.03 |
| Input variables | | | **Coded levels** | | |
|  |  |  | **Low (-1)** | **Mid (0)** | **High (+1)** |
| A | | | 6.47 | 7.74 | 9.01 |
| B | | | 0.46 | 0.69 | 0.91 |
| C | | | 0.03 | 0.10 | 0.17 |

**A: T80 (%); B: TPO (%); C: PG: T80**

**Table S2. Analysis of variance of the fitted linear, quadratic, and interactive models for output variables of TPO-NANO formulations.**

| Variables | | Droplet diameter (nm) | | | Polydispersity index (PDI) | | | |  |
| --- | --- | --- | --- | --- | --- | --- | --- | --- | --- |
|  |  | **Mean square** | **F Value** | ***p*-Value** | **Mean square** | **F Value** | | ***p*-Value** | |
| Model | | 39846 | 440.61 | 0.000^***^ | 0.020618 | 8.98 | | 0.013^*^ | |
| Linear | **A**  **B**  **C** | 9093  8826  12004 | 100.55  97.59  132.74 | 0.000^***^  0.000^***^  0.000^***^ | 0.002112  0.005513  0.000200 | 8.28  21.62  0.78 | | 0.035^*^  0.006^*^  0.416 | |
| Square | **A^2^**  **B^2^**  **C^2^** | 13770  4416  228739 | 152.26  48.83  2529.35 | 0.000^***^  0.001^**^  0.000^***^ | 0.000577  0.002792  0.000369 | 2.26  10.95  1.45 | | 0.193  0.021^*^  0.283 | |
| Interaction | **A-B**  **A-C**  **B-C** | 2601  6177  72636 | 28.76  68.31  803.19 | 0.003^**^  0.000^***^  0.000^***^ | 0.002500  0.000625  0.005625 | 9.80  2.45  22.06 | | 0.026^*^  0.178  0.005^**^ | |
| R^2^ | | 99.87% | | | 94.18% | | | |  |
| R^2^-adj | | 99.65% | | | 83.69% | | | |  |
| R^2^-pred | | 98.12% | | | 98.12%74.50% | | | |  |
| Lack of fit | | 139 | 7.86 | 0.115 | 0.000225 | 0.75 | 0.615 | |  |
| Pure Error | | 18 | | | 0.00030 | | | |  |

**A: T80 (%); B: TPO (%); C: PG: T80**


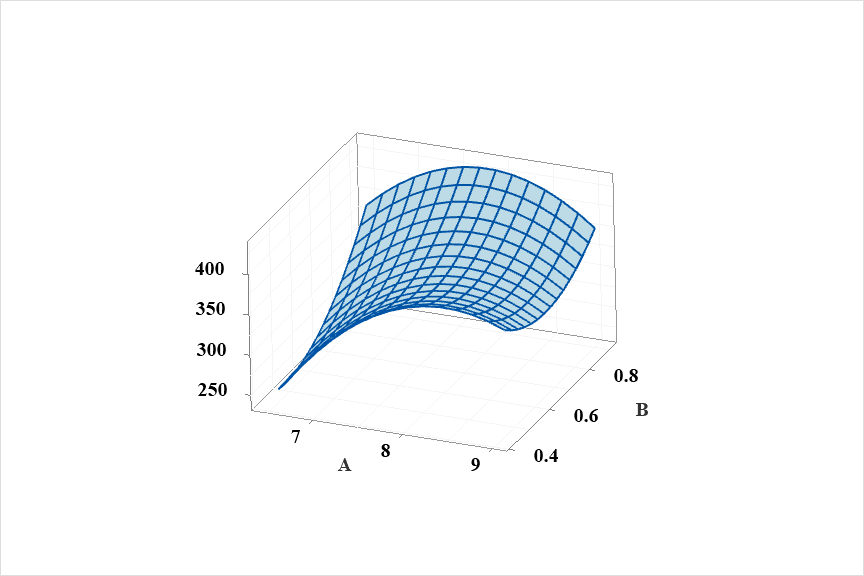

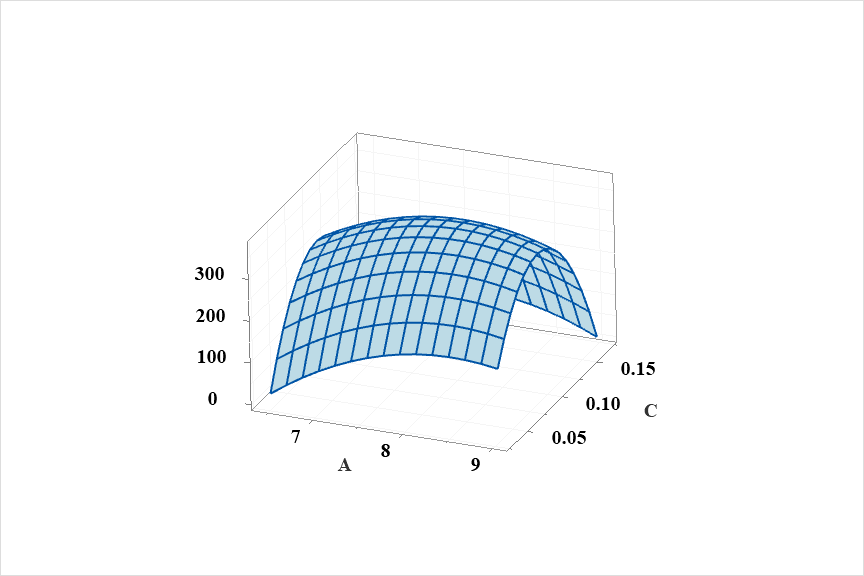

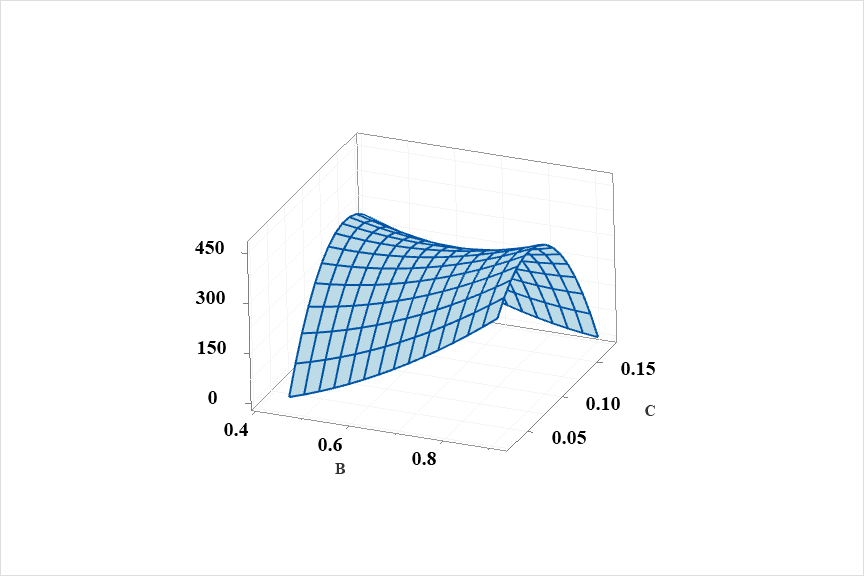

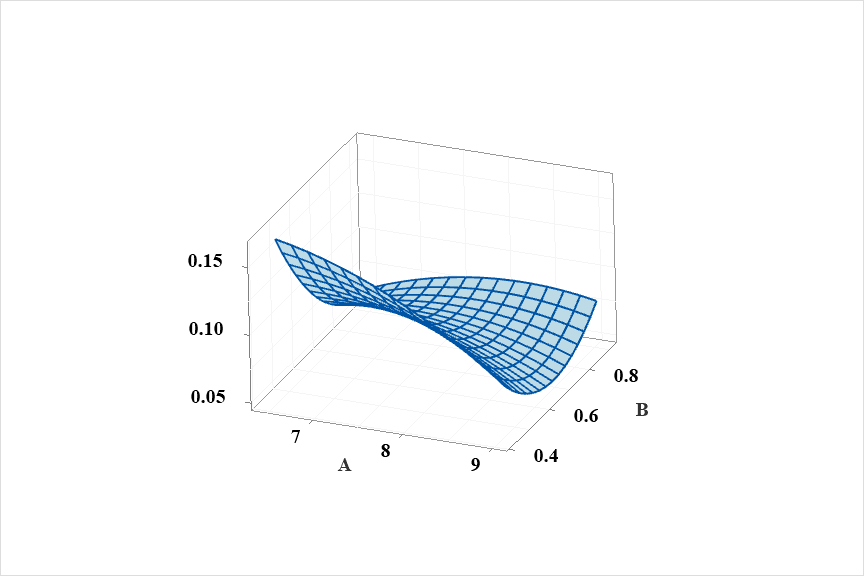

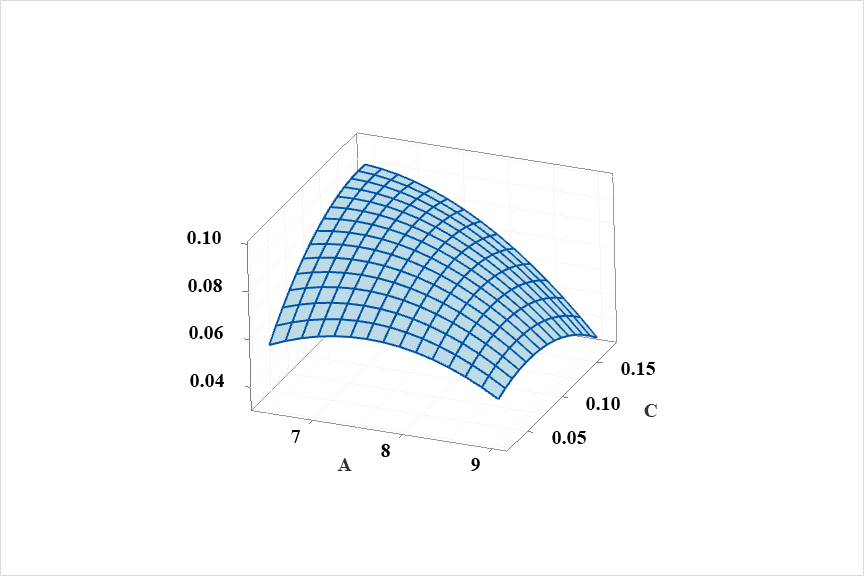

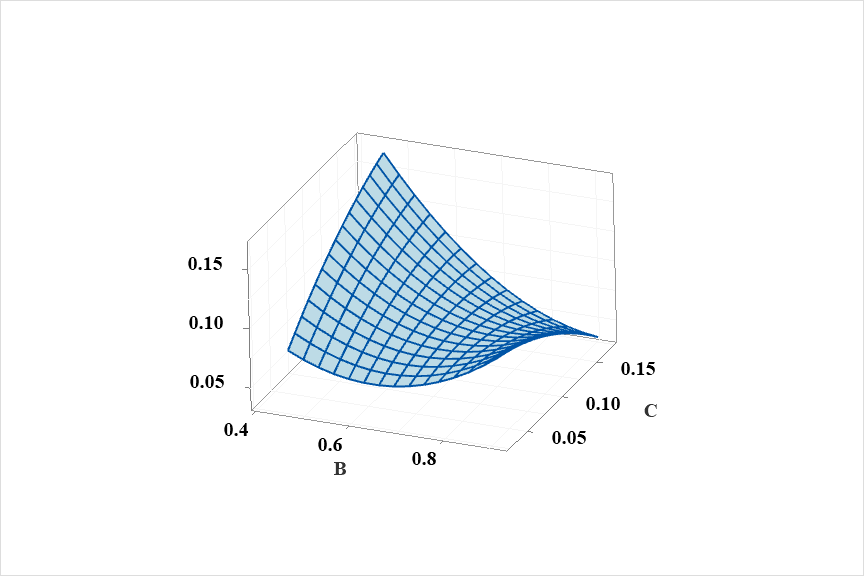


**Hold value C (0.1)**

**Hold value B (0.69)**

**Hold value A (7.74)**

**Droplet Diameter (nm)**

**PDI**

**Figure S3. Three-dimensional surface response plots showing the effects of input variables [% T80 (A) % TPO (B) and PG: T80 ratio (C)] on droplet diameter and PDI of prepared TPO-NANO showing hold-values.**

**Table S3. Comparison between predicted values and experimental values of droplet diameter and polydispersity index (PDI) of the optimized TPO-NANO.**

| **Output variables** | **Optimal value of**  **input variables** | | | **Predicted value with 95% confidence intervals** | **Experimental value**  **Mean ± sd** | **% Prediction Error ^a^** |
| --- | --- | --- | --- | --- | --- | --- |
|  | **A** | B | C |  |  |  |
| **Droplet diameter** | 7.5 | 0.9 | 0.17 | 4.63 < 12.63 < 20.63 | 12.90± 0.04 | 2.09 % |
| **PDI** |  |  |  | 0.026 < 0.0397 < 0.053 | 0.04 ± 0.009 | 1.01 % |

**^a^** $\mathbf{Error}\left( \boldsymbol{\%} \right)=\frac{Expermintal -Predicted}{\mathrm{Expermintal}}\times100$


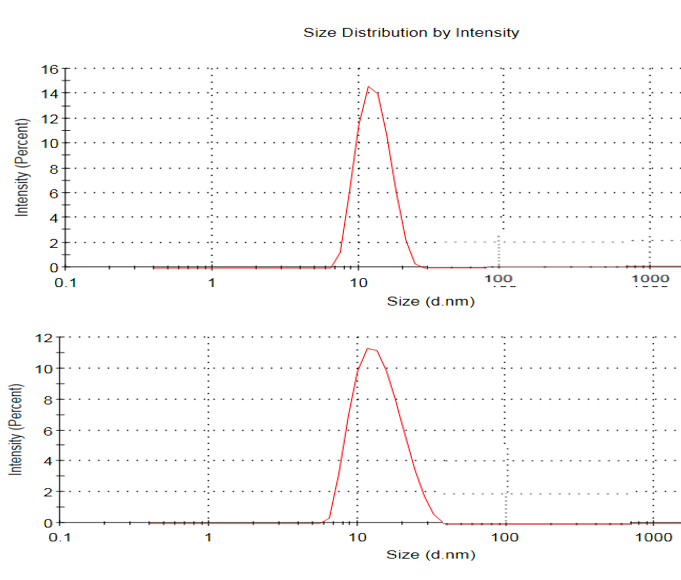


**a**

**b**

**PDI= 0.04 ± 0.009**

**PDI= 0.05 ± 0.13**

**Figure S4. Droplet diameter (nm) spectrum of TPO-NANO (A), and Oxa+TPO-NANO (B).**

**Table S4. Surviving fraction and plating efficiency of HCT116 and HT-29 cancer cell lines.**

| **Cells** | **Untreated Control**  **(mean ± sd)** | **TPO-NANO**  **(mean ± sd)** | **Oxa+TPO-NANO**  **(mean ± sd)** | **Oxa-NS**  **(mean ± sd)** |
| --- | --- | --- | --- | --- |
| **HCT116**  **SF**  **PE** | 132.02 ± 4.2 ^$$$ a,b,c^  114.9 ± 2.6 ^$$$ a,b,c^ | 56.25 ± 0.87  75 ± 2.74 | 0.0064 ± 0.002  0.8 ± 0.07 | 0.16 ± 0.06 ^***a,b^  4 ± 0.98 ^***a,b^ |
| **HT-29**  **SF**  **PE** | 11.02 ± 0.8 ^$$$ a,b,c^  33.2 ± 3.5 ^$$$ a,b^ | 1.93 ± 0.36  13.9 ± 1.87 | 2.56 ± 0.2  16 ± 1.98 | 10.11 ± 1.2 ^***a,b^  31.8 ± 3.42 ^***a,b^ |

SF: Survival fraction; PE: Plating efficiency

^a, b, c^ represents Oxa-NS, TPO-NANO and Oxa+TPO-NANO, respectively.

^$$$^ and ^***^ indicate *p* ≤ 0.0001.
